# Supplementary figures and images for: A new Neu—a syngeneic model of spontaneously metastatic HER2-positive breast cancer
Source: Clin Exp Metastasis. 2024 May 8;41(5):733–46. doi: 10.1007/s10585-024-10289-z (PMC11499368; doi:10.1007/s10585-024-10289-z)

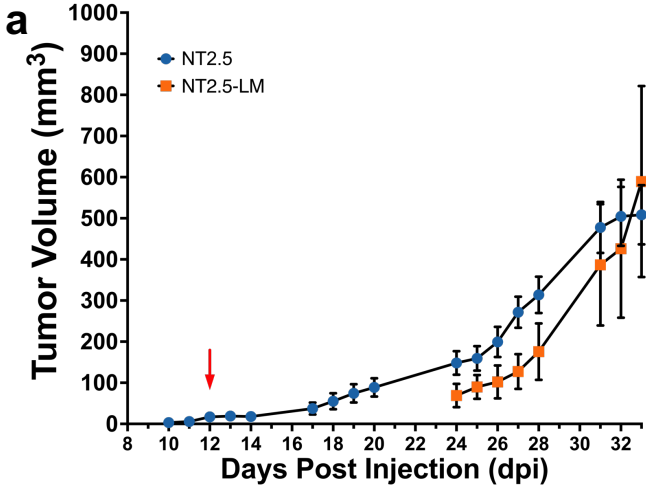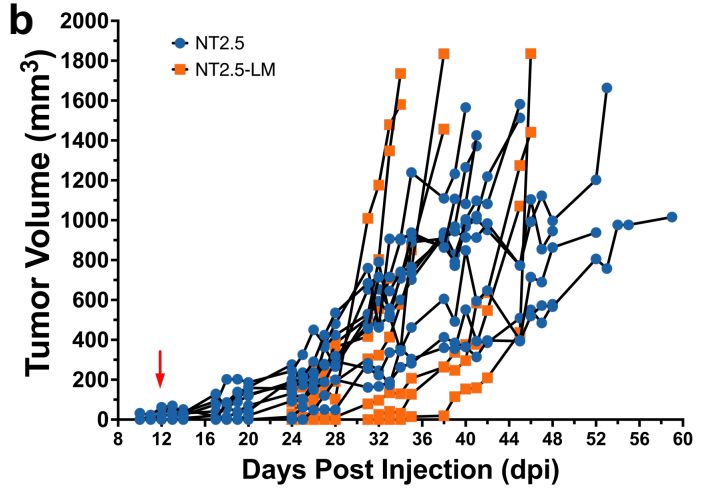

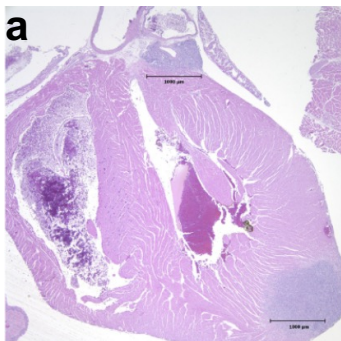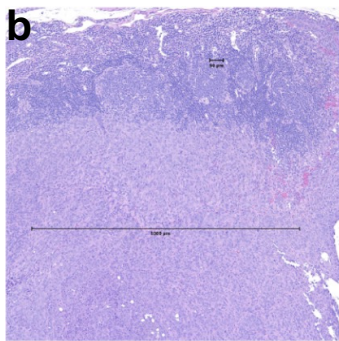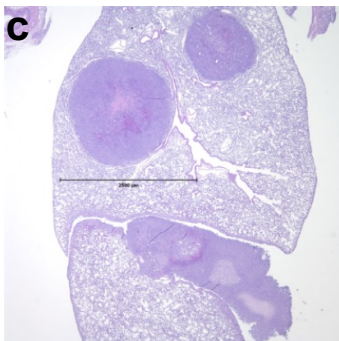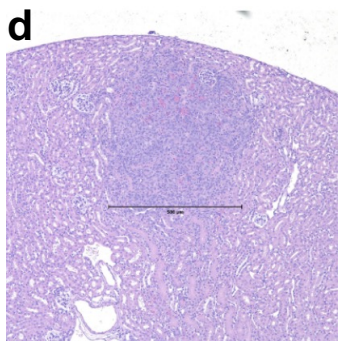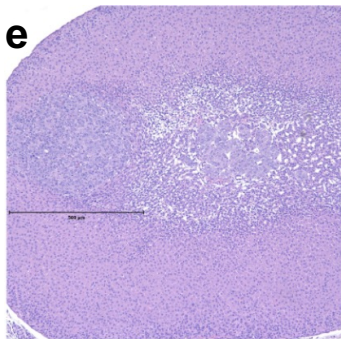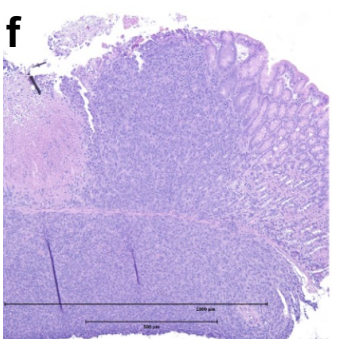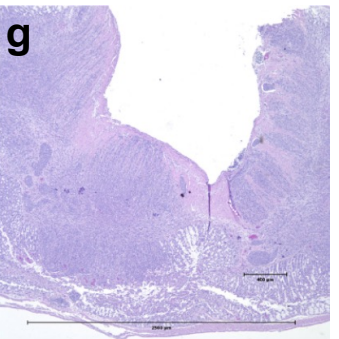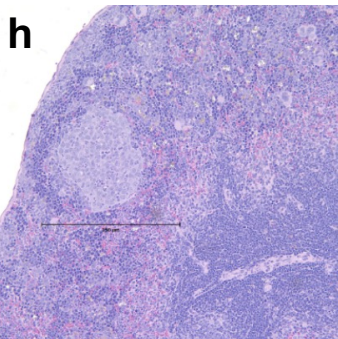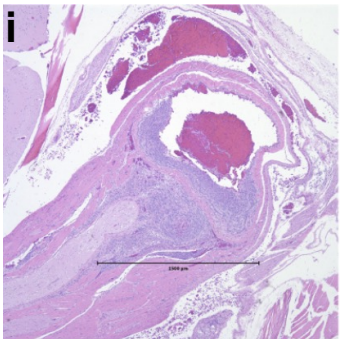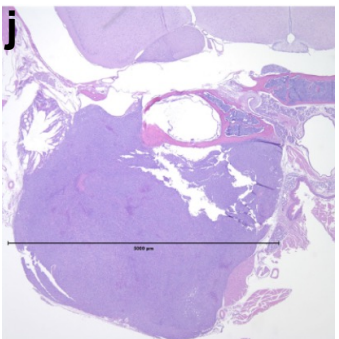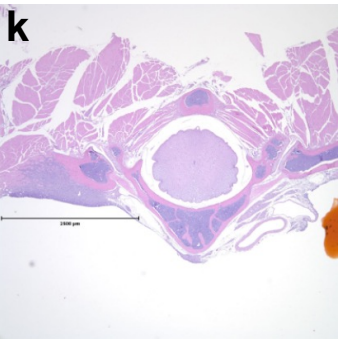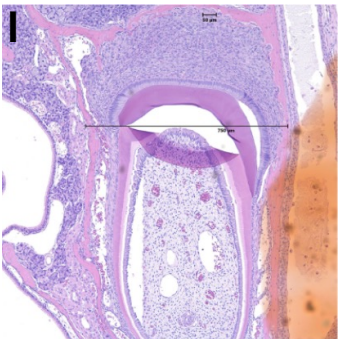

NT2.5 Breast Tumor

NT2.5-LM Lung Metastasis

EGFR

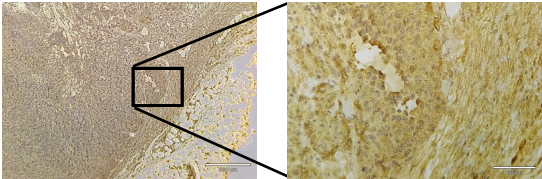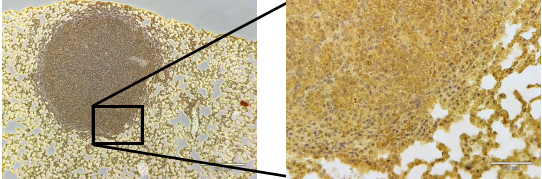

AE1/3

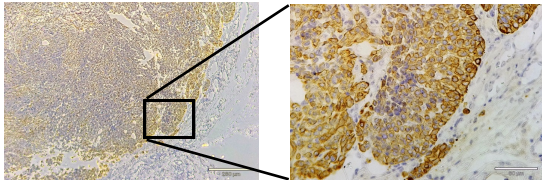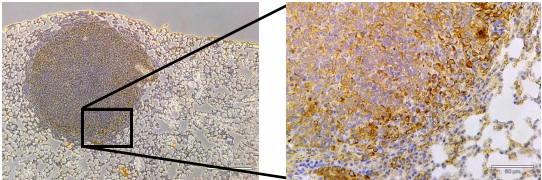

CK5

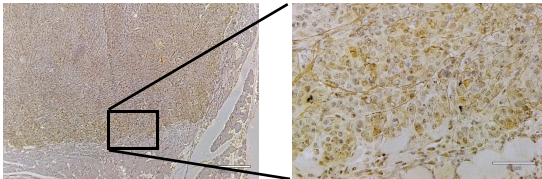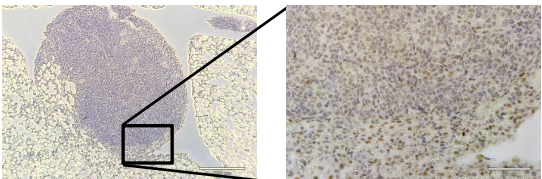

CK6

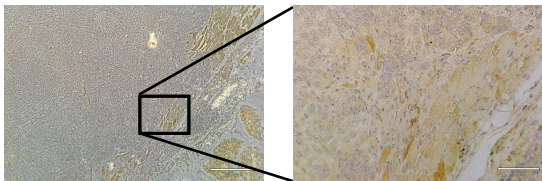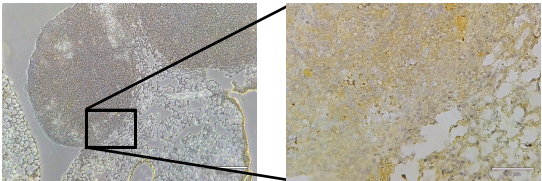

**a** Down-regulated in NT2.5-LM

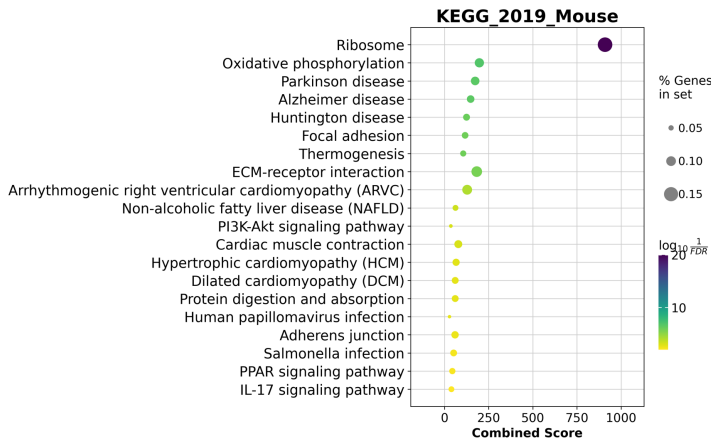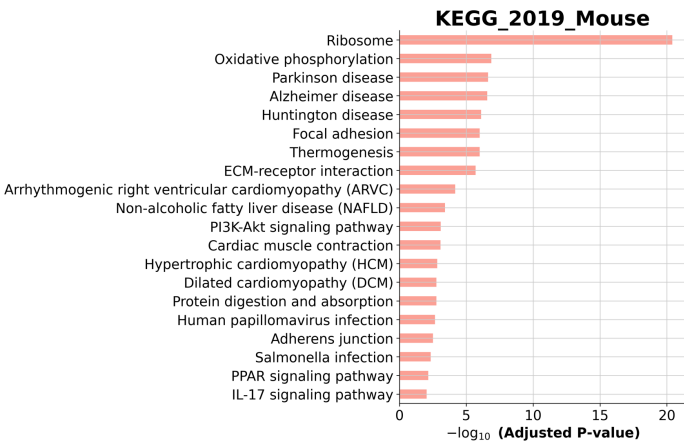

**b** Up-regulated in NT2.5-LM

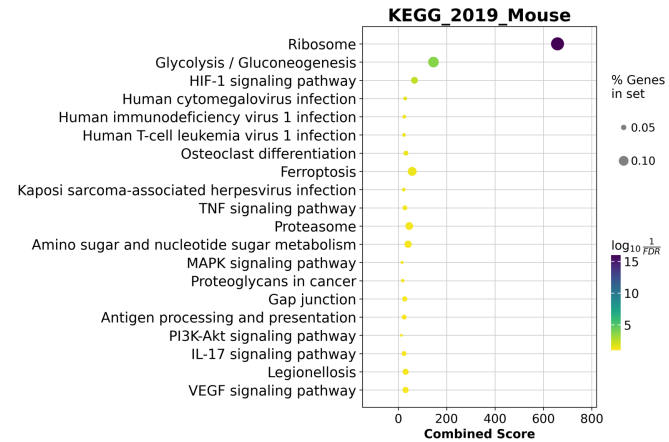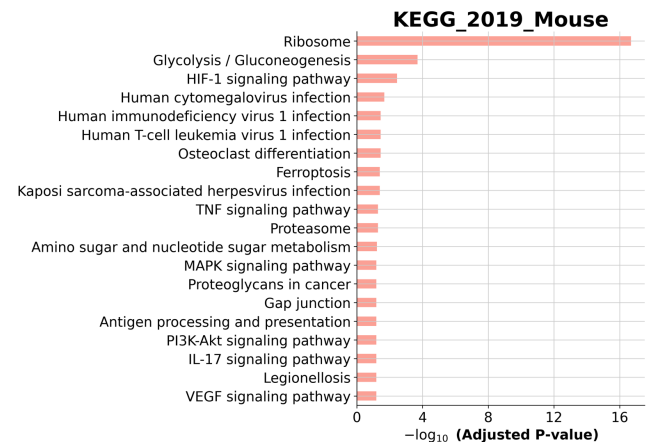

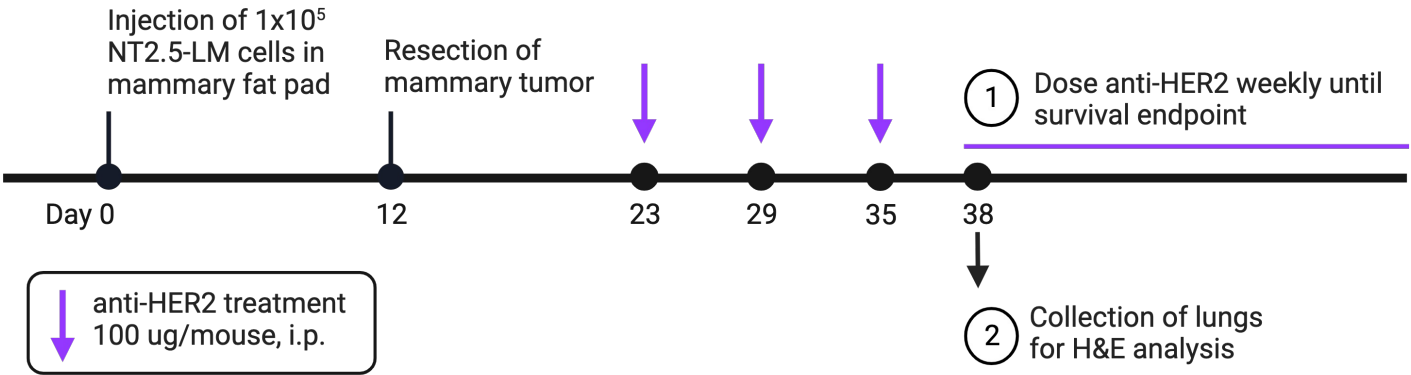

Supplement: Supplementary file 1 — Supplementary file1 (PDF 4883 KB) [file 10585_2024_10289_MOESM1_ESM.pdf]
