# Supplementary material for: A new Neu—a syngeneic model of spontaneously metastatic HER2-positive breast cancer
Source: Clin Exp Metastasis. 2024 May 8;41(5):733–46. doi: 10.1007/s10585-024-10289-z (PMC11499368; doi:10.1007/s10585-024-10289-z)
Supplement: Supplementary file 3 — Supplementary file3 (DOCX 13 KB) [file 10585_2024_10289_MOESM3_ESM.docx]

**Fig. S1 Tumor growth in NT2.5-LM model. (a)** 1x10^5^ NT2.5 or NT2.5-LM cells were injected into a mammary fat pad of NeuN mice (NT2.5, n=10; NT2.5-LM, n=7). Mammary tumor volumes (mm^3^) were averaged across all mice within the same group. Surgical resection of NT2.5-LM tumor-bearing mice at 12 days post-injection (dpi) is depicted by a red arrow. Mammary tumors regrew in NT2.5-LM at 24 dpi. Data shown until first mouse death recorded at 33 dpi. **(b)** Mammary tumor volumes (mm^3^) of individual mice shown in (a) until required euthanasia of mice.

**Fig. S2 Necropsy of NT2.5-LM metastases-bearing tissues.** Upon euthanasia of NT2.5-LM mice, various tissues were collected, fixed, sectioned, stained with H&E, and evaluated for presence of metastases. Tissues shown include **(a)** heart [scale bars: 1000 µm], **(b)** lymph nodes [scale bars: 50 µm, 1000 µm], **(c)** lungs [scale bar: 2500 µm], **(d)** kidney [scale bar: 500 µm], **(e)** adrenal gland [scale bar: 500 µm], **(f)** stomach [scale bars: 500 µm, 1000 µm], **(g)** colon [scale bars: 400 µm, 2500 µm], **(h)** spleen [scale bar: 250 µm], **(i)** skull [scale bar: 2500 µm], **(j)** ear [scale bar: 5000 µm], **(k)** body wall [scale bar: 2500 µm], and **(l)** teeth [scale bars: 50 µm, 750 µm].

**Fig. S3 Immunohistochemistry (IHC) of NT2.5 mammary tumors and NT2.5-LM lung metastases.** Staining of EGFR, AE1/3, CK5, and CK6 in NT2.5 mammary tumors (left) and NT2.5-LM lung metastases (right) collected at 35 days post-injection. Scale bars are 280 µm and 60 µm (zoomed-in panels).

**Fig. S4 Differential pathway regulation in NT2.5-LM compared to NT2.5 cancer cells**. Unsupervised pathways analysis from single cell RNA sequencing datasets by comparing top 250 differentially expressed genes with overlap in pathways from ‘KEGG_2019_Mouse’ database using Gene Set Enrichment Analysis. Top 20 pathways in NT2.5-LM that are **(a)** downregulated and **(b)** upregulated compared to NT2.5 are shown, p-adj. < 0.01. Dotted line in (b) depicts cut-off for p-adj. = 0.01.

**Fig. S5 Anti-HER2 treatment scheme for NT2.5-LM.** 1x10^5^ NT2.5-LM cells were orthotopically injected in a mammary fat pad. Mammary tumors were surgically resected 12 days post-injection (dpi). Anti-HER2 monoclonal antibody treatment of 100 µg/mouse administered intraperitoneally once a week for three weeks began at 23 dpi. After three weeks of anti-HER2 treatment, maintenance dosage for survival experiments were given once a week. For metastatic burden analysis, lungs were collected at 38 dpi for subsequent analysis.

**Table S1** **Differential pathways in NT2.5-LM compared to NT2.5 cancer cells**. All unsupervised pathways analysis from single cell RNA sequencing datasets by comparing top 250 differentially expressed genes with overlap in pathways from ‘KEGG_2019_Mouse’ database using Gene Set Enrichment Analysis.
